# Granular cell tumor mimicking a squamous cell carcinoma of the tongue: a case report

**DOI:** 10.1186/s13104-016-2325-7

**Published:** 2017-01-03

**Authors:** Jean Carlos Barbosa Ferreira, Angélica Ferreira Oton-Leite, Rafaela Guidi, Elismauro Francisco Mendonça

**Affiliations:** 1 Department of Oral Medicine (Oral Pathology), Dental School, Faculdade de Odontologia, Universidade Federal de Goiás, Praça Universitária, S/N, Setor Universitário, Goiânia-Goiás, CEP 74605-220 Brazil; 2 Department of Oral Medicine Dental, Association of Cancer of Combat of Goiás, Araujo Jorge Hospital, Goiânia, Brazil

**Keywords:** Granular cell tumor, Diagnosis, Immunohistochemistry, Squamous cell carcinoma, Case report

## Abstract

**Background:**

Granular cell tumor is a rare benign tumor that can present a pseudoepitheliomatous hyperplasia of the covering epithelium. This lesion is not encapsulated and can be characterized by a pseudo invasive growth pattern, represented by the tumoral cells that infiltrate between adjacent connective tissue elements. Diagnostic difficulties may arise because histopathological features of the pronounced pseudoepitheliomatous hyperplasia can be confused with a well-differentiated oral squamous cell carcinoma. The aim of this case report is to demonstrate the role of an immunohistochemical panel in the diagnosis of a granular cell tumor in the tongue with clinical and microscopic features resembling an oral squamous cell carcinoma.

**Case presentation:**

A 44-year-old white man with a history of heavy smoking and alcohol abuse presented an ulcerated nodular lesion in the dorsum of the tongue. The lesion was asymptomatic with fast growth. The clinical diagnosis was an oral squamous cell carcinoma. An incisional biopsy was performed and the ensuing histopathological analysis showed a pseudoepitheliomatous hyperplasia in the overlying epithelium mimicking the invasion of epithelial tumor cells into the connective tissue as in an oral squamous cell carcinoma. Immunohistochemical antibodies (S-100, vimentin, CD68, p53, Ki-67, E-cadherin, collagen IV and cytokeratin AE1/AE3) were used to characterize molecular aspects of the lesion. Strong staining of S-100 protein, CD68, vimentin, E-cadherin and low proliferative activity observed with Ki-67 expression confirmed the diagnosis of a granular cell tumor. The patient was submitted to surgical excision of the whole lesion. At a 12-month check-up, there was no evidence of recurrence.

**Conclusion:**

This case report showed that the immunohistochemical profile was helpful in determining the clinical behavior of the tumor and establishing the final diagnosis with appropriate treatment.

## Background

Granular Cell Tumor (GCT), also known as “Abrikossoff’s tumor” or “granular cell myoblastoma,” is a rare benign neoplasm of soft tissues, characterized by the accumulation of plump cells with abundant granular cytoplasm [1, 2]. GCT was described in 1926 by Abrikossoff and the etiology of this disease is controversial and much discussed in the literature [2]. Schwann cells, fibroblasts, histiocytes, myoblasts and undifferentiated mesenchymal cells have been identified in the histogenesis of GCT [2, 3].

This lesion may arise anywhere in the body, however the most common sites are in the head and neck region [4–6]. In the oral cavity, the most affected site is the tongue although, other sites such as the hard palate, buccal mucosa, lip, uvula, parotid gland and gingiva have been reported [2–6]. Some studies showed a predilection for females and occurrence in the fourth to sixth decades [2, 3].

Clinically, the lesions resemble other neoplasms such as fibromas, lipomas, neurofibromas, neuromas or schwannomas [4]. Most of the lesions are asymptomatic papules or nodules, less than 3 cm in diameter, poorly circumscribed, involving the subcutaneous or submucosal tissues [1–4]. The lesion in general is solitary, although there are reports of multicentric GCT [7].

Typical histopathological aspects of GCT include polygonal cells with small nuclei and abundant eosinophilic granular cytoplasm [3, 5]. Approximately 50% of intraoral GCT lesions present pseudoepitheliomatous hyperplasia (PEH) associated with mucosal epithelium [8]. Diagnostic difficulty arises in cases where PEH is so pronounced that it can be mistaken for a squamous cell carcinoma (SCC), especially when superficial specimens of biopsies are collected [8].

Pseudoepitheliomatous hyperplasia may occur in some lesions of the oral cavity, including granular cell tumor, median rhomboid glossitis, paracoccidioidomycosis, Wegener’s granulomatosis and necrotizing sialometaplasia [8]. The cause of PEH is unknown, but the proliferative activity of basal cells that interact with granular cells and neighboring epithelial cells can be considered a plausible explanation for the association between GCT and PEH [8].

In this specific case, we report a GCT with pronounced PEH in the tongue mimicking an oral SCC. Some studies showed that both lesions could be associated [9–12]. Therefore, an immunohistochemical panel was used as an adjunct to routine hematoxylin and eosin to help characterize molecular aspects associated with the biological behavior of this lesion.

## Case presentation

**Fig. 1:**
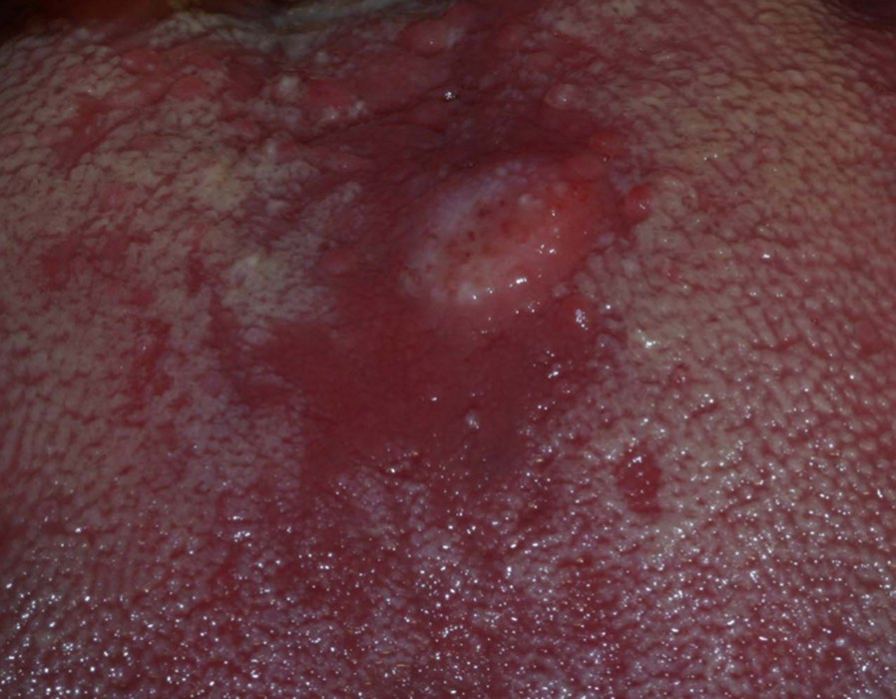
Intraoral clinical examination. Nodular lesion with erythroleukoplakia areas in the dorsum of the tongue

A 44-year-old white man was referred to the Araújo Jorge Hospital of Cancer in Brazil with a painless lesion in the dorsum of the tongue. The patient reported a history of smoking and alcohol abuse. Extraoral physical examination showed no alterations. Intraoral examination revealed an ulcerated nodular lesion with erythroleukoplakia areas, 1.5 × 1.0 cm in diameter, and rapid growth in 2 months. The clinical diagnosis was an oral SCC (Fig. 1). Written informed consent was obtained from the patient for publication of this Case Report and any accompanying images. Additionally, the authors adhered to the CARE guidelines/methodology.

**Fig. 2:**
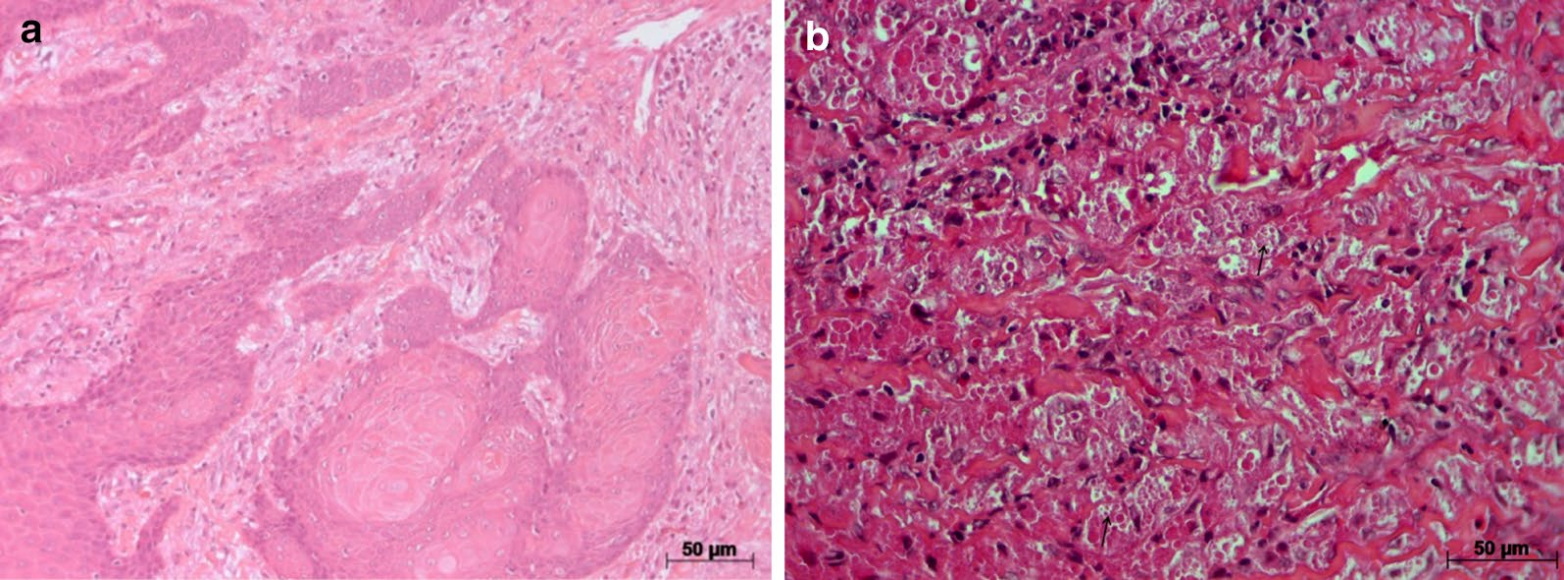
Photomicrograph of a granular cell tumor with severe pseudoepitheliomatous hyperplasia and granular cells in **a** (hematoxylin and eosin, original magnification 100×). **b** Histopathological aspect of granular cell tumor, characterized by the presence of granular cytoplasm cells (*arrows*) and round nuclei (hematoxylin and eosin, original magnification 400×)

An incisional biopsy was performed and the specimen was fixed in 10% buffered formalin and submitted to histopathological analysis. The microscopic findings stained by hematoxylin and eosin showed a PEH in the overlying epithelium mimicking the invasion of epithelial tumor cells into the connective tissue as in oral SCC (Fig. 2a). However, in a high field of view, the lesion was composed of cells with large eosinophilic, granular cytoplasm and small hyperchromatic central nuclei, characteristics of a GCT (Fig. 2b).

**Fig. 3:**
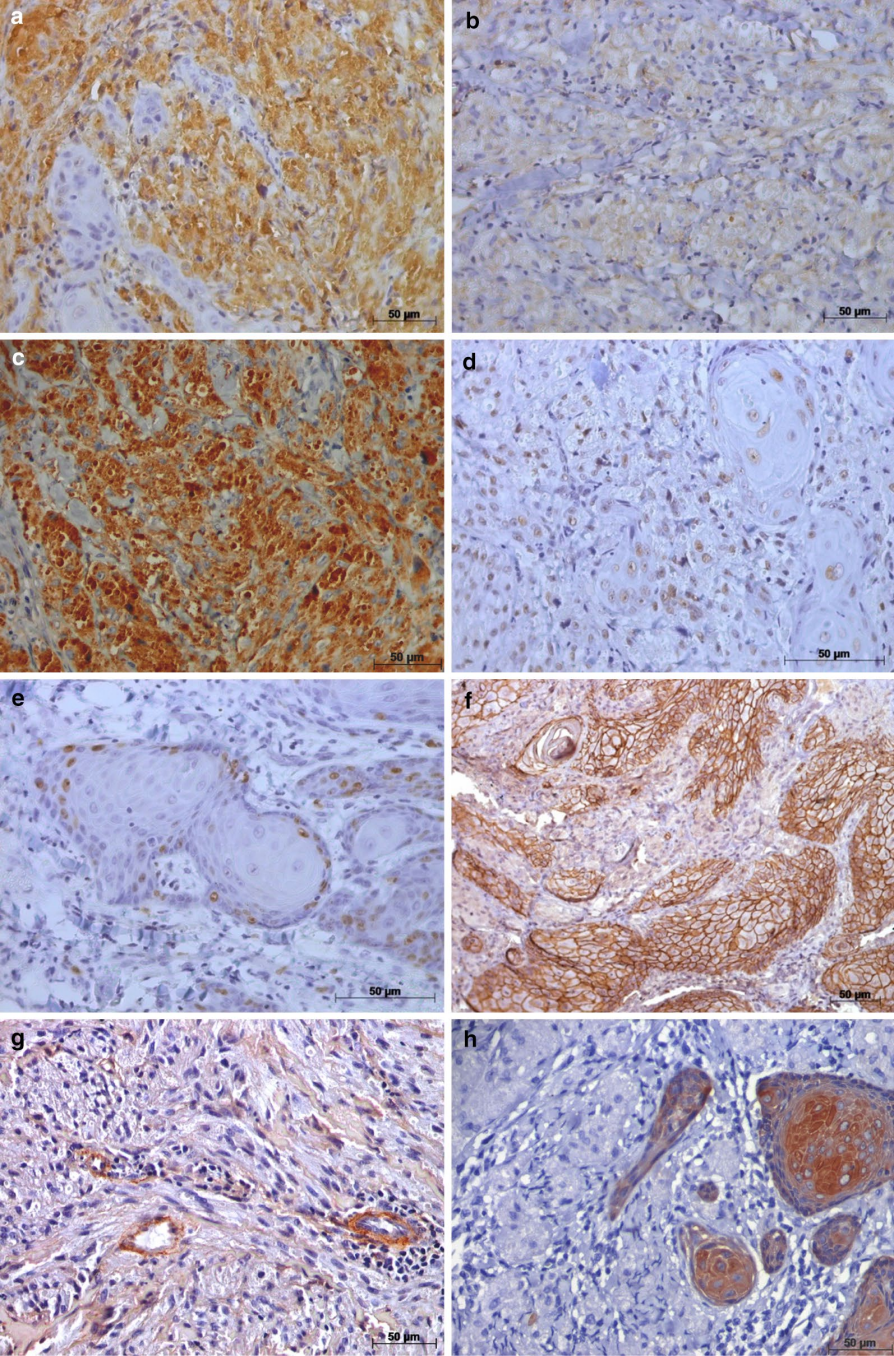
Photomicrograph of immunohistochemical staining of granular cell tumor: **a** strong nuclear and cytoplasm staining of S-100 protein (S-100, original magnification 400×); **b** granular cell staining of vimentin in cytoplasm (vimentin, original magnification 400×); **c** strong staining in the cytoplasm of granular cells for CD68 (CD68, original magnification 400×); **d** nuclear staining of granular cells for p53 (p53, original magnification 400×); **e** Ki-67 immunostaining in the basal and parabasal cells of epithelium and some isolated granular cells (Ki-67, original magnification 400×); **f** membranous staining in the overlying epithelium for E-cadherin (E-cadherin, original magnification 200×); **g** immunostaining collagen IV present only in the endothelium (collagen IV, original magnification 400×); **h** negative expression of cytokeratin in granular cells (cytokeratin AE1/AE3, original magnification 400×)

**Fig. 4:**
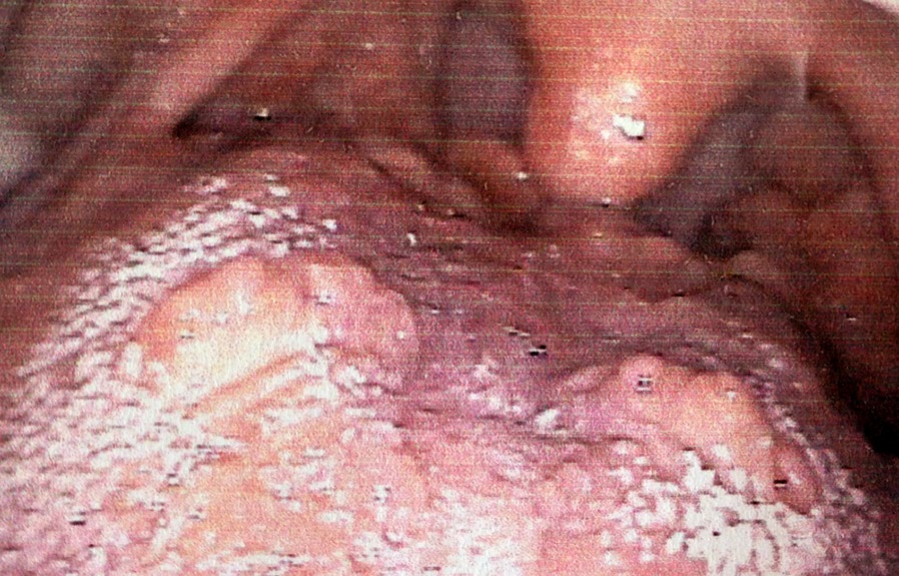
A postsurgical intraoral view

The immunohistochemical panel was fundamental in determining biological aspects of the lesion and the final diagnosis was GCT with PEH (Fig. 3). The patient was submitted to surgical excision of the whole lesion and no recurrence was noted (Fig. 4). Furthermore, it was recommended that the patient stop smoking and drinking.

Based on the microscopic findings, an immunohistochemical panel using the Streptavidin–Biotin complex technique was performed. The specific antibodies used were S-100, vimentin, CD68, p53, Ki-67, E-cadherin, collagen IV and cytokeratin AE1/AE3. The dilutions and main information about these markers are described in Table 1.

**Table 1:** Characteristics of immunohistochemical markers

Antibodies	Clone	Source	Dilution	Antigen retrieval	Control tissue
S-100	Z0311	Dako^®^	1:1000	Citrate, pH 6.0	Melanoma
Vimentin	J144	Santa Cruz^®^	1:100	Citrate, pH 6.0	Lymph node
CD68	KP1	Novocastra^®^	1:1000	Citrate, pH 6.0	Liver
p53	DO-7	Novocastra^®^	1:200	Citrate pH 6.0	Squamous cell carcinoma
Ki-67	MM1	Novocastra^®^	1:100	Citrate, pH 6.0	Squamous cell carcinoma
E- cadherin	NCH-8	Dako^®^	1:400	Citrate, pH 6.0	Mammary ductal carcinoma
Collagen IV	CIV22	Dako^®^	1:100	Trypsin, pH 7.0	Kidney
Cytokeratin	AE1/AE3	Dako^®^	1:1000	Citrate, pH 6.0	Breast

Our results showed a strong nuclear and cytoplasmic staining of S-100 protein and of vimentin in the cytoplasm of the polygonal cells of GCT (Fig. 3a, b). CD68 revealed a strong staining in the cytoplasm of granular cells (Fig. 3c). The expression of p53 was found in the nuclei of basal and parabasal cells of normal epithelium, as well as a diffuse staining in the granular cells and PEH (Fig. 3d). In addition, cells of the basal layer of the epithelium were weakly marked with Ki-67 (less than 10%), mainly in the PEH and some isolated granular cells (Fig. 3e). E-cadherin showed a uniform “membranous staining” in the overlying epithelium (Fig. 3f). Some regions of the endothelium were marked with collagen IV (Fig. 3g). Finally, granular cells were negative for cytokeratin AE1/AE3 (Fig. 3h). The immunohistochemical staining of these markers is summarized in Table 2.

**Table 2:** Immunohistochemical results

Antibody	Results	Staining site
S-100	+++	Nuclear and cytoplasm of granular cells
Vimentin	+++	Cytoplasm of granular cells
CD68	+++	Cytoplasm of the granular cells
p53	++	Nuclear staining of the basal and parabasal layer of normal epithelium, granular and pseudoepitheliomatous hyperplasia cells
Ki-67	+	Nuclei of basal and parabasal layer and some isolated granular cells
E-cadherin	+++	Membranous staining in pseudoepitheliomatous hyperplasia cells
Collagen IV	++	Cytoplasm of endothelium cells
Cytokeratin AE1/AE3	+++	Cytoplasm of pseudoepitheliomatous hyperplasia cells

+ weak, ++ moderate, and +++ strong

## Conclusions

GCT is a benign tumor that occasionally induces PEH of the covering epithelium, mimicking an invasive oral SCC, which can make diagnosis difficult in some cases [2, 4]. However, some studies have reported the coexistence of the two lesions in the dorsum of the tongue and another one showed that is also a common site of an oral SCC [9–13]. For this reason, and given the history of heavy smoking, alcoholism and the fast growth of the tongue lesion, it was important to investigate the neoplastic potential of GCT using the immunohistochemical panel of S-100, vimentin, CD68, p53, Ki-67, E-cadherin, collagen IV and cytokeratin AE1/AE3 antibodies.

Several immunohistochemistry studies have been conducted to investigate the origin of GCT [1–3], while others have investigated its clinical behavior and possible association between this neoplasm and other malignant lesions in the oral cavity [4, 5, 9–12]. Most authors consider that Schwann cells are the precursors of GCT because of positivity for S-100 and vimentin [1, 2, 4]. In line with the literature, immunohistochemical analysis of S-100 and vimentin in the present case revealed strong and diffuse staining in GCT [1, 2]. This result reinforces the diagnosis of GCT.

With regard to S-100 and vimentin expressions in oral SCC, Albuquerque et al. [14] found an increased number of reactive cells for S-100 protein in dendritic cells from the tumor site. Furthermore, vimentin expression has frequently been detectable in the majority of finger-like invasive fronts of tumors and may be associated with the metastatic conversion of epithelial cells and tumor invasion [15]. In our clinical case, the expression of vimentin was negative in epithelial cells of GCT.

Another common immunohistochemical marker that has been traditionally used for identifying GCT is CD68, a marker of lysosomes, mostly associated with macrophages, which is also usually positive in GCT [2, 16], as shown in the present case. In contrast, the immunohistochemical staining of CD68 in oral SCC was distributed diffusely along the inflammatory infiltrate [17].

It is important to emphasize that few studies have investigated the expression of p53 protein, an important tumor suppressor gene related to oral carcinogenesis, in benign GCT [18–20]. Caltabiano et al. [10] evaluated the expression of oncoprotein p53 in a case of GCT and SCC colocalized at the same site. They showed that immunohistochemical reactivity for p53 was increased within the nuclei of the invasive tumor cells in the full thickness of the epithelium as found by Zarovnaya et al. [18] in SCC. According to our findings, Zarovnaya et al. [18] showed that nuclear staining of p53 was limited to the basal cells and the cells adjacent in a linear pattern in cases of benign mucosa with varying degrees of PEH. Furthermore, p53 protein accumulation can be found in benign lesions and may represent a response to cellular stresses [19].

The immunostaining of Ki-67 in GCT showed a very low number of positive cells in basal and parabasal layers and some isolated granular cells [8, 16, 19]. Coincidentally, in cases of oral SCC the basal and parabasal layers also exhibit staining of Ki-67. However, high Ki-67 expression levels of neoplastic cells at the invasive tumor front are noted [17, 20]. Thus, the increase of Ki-67 immunostaining in cases of GCT could be considered an important signal of the predilection of tumor behavior and may suggest a possible association with other malignant lesions such as SCC.

With regard to E-cadherin and collagen IV expressions, we found a uniform membranous staining of E-cadherin, an intercellular calcium-dependent cell adhesion molecule, in the overlying epithelium. This is in accordance with the study of Zarovnaya et al. [18], which showed no loss of cell adhesion in benign PEH. This same study revealed decreased or absent staining for E-cadherin in malignant cell clusters in oral SCC. In addition, the main component of basement membrane, collagen IV, was absent in our case as found in benign inflamed areas within PEH [18]. Therefore, our results also suggested that collagen IV is not useful for differentiating PEH of SCC. Another important molecular marker for characterizing GCT was cytokeratin AE1/AE3, which showed negative in granular cells.

The association of clinical data with immunohistochemical findings was essential to confirm the diagnosis of the lesion. Neither cellular atypia, pleomorphism, necrosis, high mitotic index or other histological signs of malignancy were identified in our case. However, in cases where there is doubt, the immunohistochemical panel could be useful for excluding characteristics of a malignant neoplasm and avoiding an aggressive therapy.

The aim of this case report was to use the immunohistochemistry panel as a tool complementary to hematoxylin and eosin to help distinguish invasive SCC of GCT-PEH (granular cell tumor-pseudoepitheliomatous hyperplasia), especially when risk factors such as alcohol and tobacco are associated. It is important to emphasize that a characteristic microscopic finding of GCT is the presence of varying degrees of pseudoepitheliomatous hyperplasia of the overlying epithelium. Our results showed that GCT behaves as a benign neoplasia, but tumors with rapid growth, high Ki-67 and p53 expressions should be viewed with caution and require a long-term follow-up.

## Abbreviations

GCT: granular cell tumor
GCT-PEH: granular cell tumor-pseudoepitheliomatous hyperplasia
PEH: pseudoepitheliomatous hyperplasia
SCC: squamous cell carcinoma
